# Gastrointestinal Diagnostic Coding After Spinal Cord Injury: Health Behavior Correlates and Implications for Neurogenic Bowel Management in a Nationwide Claim-Based Cohort

**DOI:** 10.3390/jcm15020760

**Published:** 2026-01-16

**Authors:** Young-Hwan Lim, Jae-Hyeong Yoo, Jeong-Won Park, Jong-Moon Hwang, Dongwoo Kang, Jungkuk Lee, Hyun Wook Han, Kyung-Tae Kim, Myung-Gwan Kim, Tae-Du Jung

**Affiliations:** 1 Department of Rehabilitation Medicine, Kyungpook National University Hospital, Daegu 41944, Republic of Korea; limyoungh@naver.com (Y.-H.L.); wtiger0308@naver.com (J.-H.Y.); xhrl0904@naver.com (J.-W.P.); 2 Department of Rehabilitation Medicine, Happy Rehabilitation Medicine Clinic, Daegu 42122, Republic of Korea; 3 Department of Data Science, Hanmi Pharmaceutical Co., Ltd., Seoul 05545, Republic of Korea; dongwoo.kang94@hanmi.co.kr (D.K.); jungkuk.lee@hanmi.co.kr (J.L.); 4 Department of Biomedical Informatics, Graduate School of Medicine, CHA University, Seongnam 13488, Republic of Korea; stepano7@gmail.com; 5 Institute for Biomedical Informatics, Graduate School of Medicine, CHA University, Seongnam 13488, Republic of Korea; 6 Department of Neurosurgery, Bokwang Hospital, Daegu 42734, Republic of Korea; nskimkt7@gmail.com; 7 Joint Institute for Regenerative Medicine, Kyungpook National University Hospital, Daegu 41944, Republic of Korea; 8 Department of Rehabilitation Medicine, School of Medicine, Kyungpook National University, Daegu 41944, Republic of Korea; 9 Department of Rehabilitation Medicine, Kyungpook National University Chilgok Hospital, Daegu 41404, Republic of Korea

**Keywords:** spinal cord injury, gastrointestinal diagnostic codes, neurogenic bowel dysfunction, physical activity, nationwide cohort study

## Abstract

**Background**: Neurogenic bowel dysfunction (NBD) is a major chronic sequela of spinal cord injury (SCI) with substantial implications for rehabilitation and long-term management. However, population-level evidence describing how gastrointestinal (GI) diagnostic codes are used following SCI, particularly within administrative healthcare systems, remains limited. **Methods**: We conducted a nationwide retrospective cohort study using administrative claims data from the Korean National Health Insurance Service (NHIS). A total of 584,266 adults with trauma-related SCI encounters between 2009 and 2019 were identified. GI diagnostic codes—paralytic ileus (K56), irritable bowel syndrome (K58), and functional bowel disorders (K59)—were evaluated as administrative proxies for bowel dysfunction. Demographic characteristics, disability status, regional factors, and health behaviors were analyzed using multivariable logistic regression. **Results**: GI diagnostic codes were frequently recorded after SCI, most commonly irritable bowel syndrome (approximately 30%) and functional bowel disorders (approximately 37%), whereas paralytic ileus was uncommon. Greater disability severity, female sex, older age, and rural residence were consistently associated with higher odds of GI diagnostic coding. Physical activity showed robust inverse associations across all models. Inverse associations observed with smoking and alcohol consumption were interpreted as reflecting residual confounding or health-related selection, rather than biological protective effects. **Conclusions**: Patterns of GI diagnostic coding after SCI likely reflect the clinical burden and management needs of neurogenic bowel dysfunction within healthcare systems, rather than the development of new gastrointestinal diseases. These findings underscore the importance of individualized bowel management, incorporation of structured physical activity into rehabilitation programs, and equitable access to SCI rehabilitation services, particularly for individuals with greater disability or those living in rural areas.

## 1. Introduction

Spinal cord injury (SCI) arises from both traumatic causes, such as vertebral fractures, and non-traumatic etiologies, including infection, vascular injury, or degenerative conditions [1]. Globally, the absolute incidence, prevalence, and years lived with disability (YLD) attributable to SCI increased between 1990 and 2019, largely driven by population growth and aging, despite declining age-standardized incidence and prevalence rates over the same period [2]. Epidemiological studies have consistently shown that older adults bear a disproportionate burden of SCI-related disability, reflecting both injury mechanisms and age-related vulnerability [3].

SCI results in profound neuromuscular and autonomic dysfunction across multiple organ systems. Among these, bowel impairment—collectively termed neurogenic bowel dysfunction (NBD)—represents one of the most prevalent and burdensome chronic sequelae, substantially affecting quality of life, independence, and long-term rehabilitation outcomes [4,5]. Individuals with SCI commonly experience constipation, abdominal distension, abdominal discomfort, and delayed colonic transit, symptoms that often persist for years after injury and require ongoing clinical management [4,6].

Importantly, NBD is a functional syndrome rather than a discrete gastrointestinal disease, and its clinical manifestations are not directly captured by a single diagnostic entity within the International Classification of Diseases (ICD) framework. In routine clinical practice, bowel-related symptoms following SCI are therefore frequently documented using gastrointestinal (GI) diagnostic codes such as paralytic ileus (K56), irritable bowel syndrome (K58), and other functional bowel disorders (K59). Within administrative healthcare databases, these codes tend to function as administrative proxies for bowel symptom burden and management needs, rather than as indicators of newly developed gastrointestinal pathology.

Health-related behaviors—including physical activity, smoking, alcohol consumption, and body mass index (BMI)—have been associated with bowel symptoms and healthcare utilization in the general population [7,8,9]. Similar behavioral and functional factors may influence symptom reporting, healthcare-seeking behavior, or diagnostic coding practices among individuals with SCI. Although several studies from Western populations have examined bowel dysfunction after SCI, population-based evidence from Asian countries remains comparatively limited, despite differences in healthcare systems, rehabilitation access, and coding practices [10,11]. In South Korea and other East Asian settings, SCI epidemiology and long-term functional outcomes have often been described using registry or claim-based data, underscoring the importance of understanding how administrative diagnostic codes are applied in real-world clinical contexts.

Accordingly, using nationwide administrative claims data from South Korea, this study aimed to (1) describe patterns of GI-related diagnostic coding following trauma-related SCI and (2) examine demographic, disability-related, regional, and health behavior correlates of these coding patterns. Throughout the analysis, GI diagnostic codes were interpreted not as evidence of new-onset gastrointestinal disease, but as markers of healthcare encounters related to the clinical burden and management of neurogenic bowel dysfunction. Understanding these coding patterns may also inform rehabilitation planning, resource allocation, and population-level assessment of bowel-related care needs in individuals with SCI.

## 2. Materials and Methods

### 2.1. Data Source and Study Population

**Figure 1:**
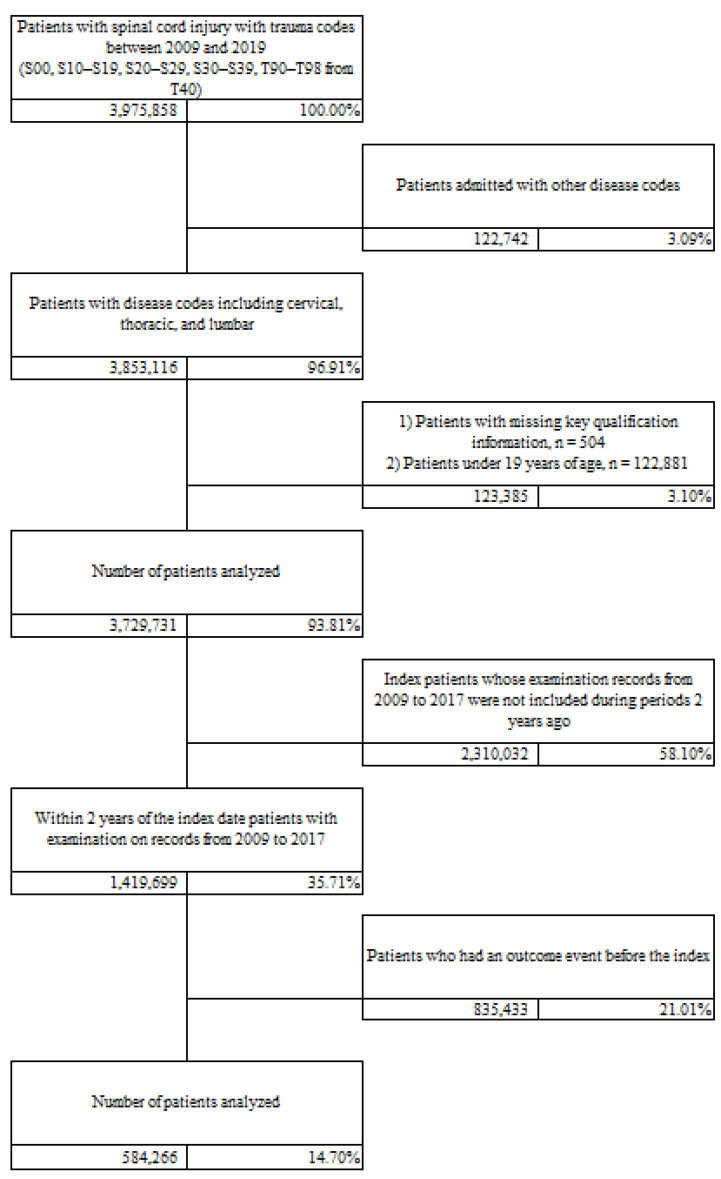
Data Collection Process. A nationwide claim-based cohort from the National Health Insurance Service (NHIS) was used to identify individuals with trauma-related spinal cord injury-associated healthcare encounters between 2009 and 2019. Of 3,975,858 individuals initially identified using relevant KCD codes, exclusions were applied sequentially for unrelated diagnoses, missing data or age < 19 years, insufficient follow-up (<2 years), and indeterminate outcomes. The final analytical cohort consisted of 584,266 individuals. Outcome events included paralytic bowel obstruction without hernia (ICD-10: K56), irritable bowel syndrome (ICD-10: K58), and other functional bowel disorders (ICD-10: K59).

Data were obtained from the National Health Insurance Service (NHIS) database of South Korea, using a nationwide claim-based cohort covering the years 2009–2019 [12]. To identify individuals with trauma-related spinal cord injury-associated healthcare encounters, we applied an operational definition based on Korean Standard Classification of Diseases (KCD) codes corresponding to traumatic injuries involving the head, neck, thorax, abdomen, and spine (S00, S10–S19, S20–S29, S30–S39), as well as sequelae of injury (T90–T98). This approach was intended to capture real-world trauma-related SCI encounters within administrative data, rather than clinically adjudicated SCI diagnoses. Therefore, our findings should be interpreted as reflecting healthcare utilization patterns among individuals with trauma-related SCI-associated encounters, rather than precise epidemiological estimates of clinically confirmed SCI. Among 3,975,858 individuals initially identified using these criteria, 122,742 were excluded due to unrelated disease codes, and 123,385 were excluded because of missing data or age under 19 years. To ensure sufficient follow-up for the assessment of post-injury gastrointestinal diagnostic coding, we further excluded 2,310,032 individuals who were not followed for at least two years after the index injury and 853,433 individuals with indeterminate outcome data. After applying these exclusion criteria, a total of 584,266 individuals were included in the final analytical cohort (Figure 1).

### 2.2. Gastrointestinal Diagnostic Codes

Gastrointestinal (GI) outcomes were operationalized using ICD-10 codes that are commonly recorded in administrative claims data during healthcare encounters related to bowel symptoms after spinal cord injury, including paralytic ileus (K56), irritable bowel syndrome (K58), and other functional bowel disorders (K59). These codes were not intended to represent discrete gastrointestinal disease entities, but were interpreted as administrative proxies for bowel symptom-related healthcare utilization and management needs in individuals with SCI. GI diagnostic codes were ascertained only after the index SCI-related encounter, and codes recorded prior to the index date were not included in the analysis.

### 2.3. Covariates

Demographic variables included age, sex, and residential area, categorized as metropolitan area, metropolitan city, or rural. Disability severity was classified using nationally registered disability grades, which reflect functional impairment levels within the Korean social security system. Health behavior-related variables were obtained from claim-linked NHIS health examination data and included smoking status, alcohol consumption frequency, body mass index (BMI), and self-reported levels of physical activity (vigorous, moderate, and walking). These health behavior variables were treated as baseline behavioral proxies for adjustment purposes, rather than as direct measures of causal effects. BMI categories were defined according to Asian-specific criteria recommended by the World Health Organization.

### 2.4. Statistical Analysis

Descriptive analyses were performed to summarize participant characteristics. Chi-squared tests were used to examine differences in gastrointestinal diagnostic coding across categories of disability severity. Multivariable logistic regression models were applied to estimate adjusted odds ratios (ORs) and 95% confidence intervals (CIs) for associations between demographic and health behavior-related covariates and each GI diagnostic code group. Outcomes were analyzed as binary indicators of occurrence within five years to capture clinically relevant healthcare encounters, acknowledging that this approach does not reflect frequency or chronicity of symptoms. Formal a priori sample size or power calculations were not performed, as this study used a fixed, population-based administrative cohort; however, the large sample size provided sufficient power to detect even modest associations. Given the large sample size, statistical significance was interpreted with caution, and effect sizes were considered alongside *p*-values. A two-sided *p*-value < 0.05 was considered statistically significant. All analyses were conducted using SAS Enterprise Guide version 7.1 (SAS Institute Inc., Cary, NC, USA) [13].

## 3. Results

### 3.1. Baseline Characteristics by Injury Level

Table 1 summarizes the baseline characteristics of participants stratified by injury level. Across all injury levels, participants were predominantly male, and the largest proportion belonged to the middle-aged group (46–64 years). Most individuals resided in rural areas, followed by metropolitan cities and the capital region.

Disability severity, classified using the national physical disability grading system, varied by injury level, with higher proportions of severe disability observed among individuals with thoracic-level injuries compared with cervical or lumbar levels. With respect to health-related characteristics, most participants had body mass index values within the normal or overweight ranges, while underweight status was uncommon.

Overall patterns of smoking status, alcohol consumption, and physical activity were broadly similar across injury levels, with low engagement in vigorous or moderate physical activity and relatively higher participation in walking.

### 3.2. Frequency of Gastrointestinal Diagnostic Coding by Injury Level

Table 2 presents the frequency of gastrointestinal diagnostic coding within five years following trauma-related spinal cord injury-associated encounters, stratified by injury level. Diagnostic coding for paralytic ileus was uncommon across all groups. In contrast, diagnostic codes for irritable bowel syndrome and other functional bowel disorders were frequently recorded, with similar proportions observed across cervical, thoracic, and lumbar injury levels.

Overall, functional bowel-related diagnostic codes were substantially more prevalent than paralytic ileus-related codes, regardless of injury level.

### 3.3. Factors Associated with Paralytic Ileus by Injury Level

Table 3 presents the results of multivariable logistic regression analyses examining factors associated with diagnostic coding for paralytic ileus within five years following trauma-related spinal cord injury-associated encounters, stratified by injury level.

Across cervical, thoracic, and lumbar injury levels, disability severity emerged as the most consistent factor associated with paralytic ileus, with both mild and severe disability showing substantially higher odds compared with no registered disability. Older age, particularly ≥65 years, was also associated with increased odds, most prominently among individuals with lumbar-level injuries. In addition, residence in rural or provincial areas was consistently associated with higher odds of paralytic ileus across injury levels.

In contrast, physical activity demonstrated consistent inverse associations, with vigorous activity, moderate activity, and regular walking all associated with lower odds of paralytic ileus. Smoking and alcohol consumption were also inversely associated with paralytic ileus; however, these associations were modest in magnitude and were interpreted cautiously.

Although many associations reached statistical significance, several effect sizes were close to unity, suggesting limited clinical relevance despite statistical significance in this large sample.

### 3.4. Factors Associated with Irritable Bowel Syndrome by Injury Level

Table 4 summarizes the results of multivariable logistic regression analyses examining factors associated with the occurrence of irritable bowel syndrome (IBS) within five years following trauma-related spinal cord injury-associated encounters, stratified by injury level.

Across all injury levels, female sex, older age, and disability severity were consistently associated with higher odds of irritable bowel syndrome. The magnitude of these associations was modest but directionally consistent across injury levels.

Physical activity again showed uniform inverse associations, suggesting an association with lower odds of IBS among individuals engaging in vigorous or moderate activity or regular walking. Smoking and alcohol consumption were inversely associated with IBS; however, these associations were small in effect size and likely reflect residual confounding, differences in health status, or healthcare-seeking behavior rather than biologically protective effects.

Overall, demographic and functional factors demonstrated more consistent and clinically interpretable associations than health behavior-related variables.

### 3.5. Factors Associated with Other Functional Bowel Disorders by Injury Level

Table 5 presents the results of multivariable logistic regression analyses examining factors associated with diagnostic coding for other functional bowel disorders within five years following trauma-related spinal cord injury-associated encounters, stratified by injury level.

Patterns observed for other functional bowel disorders were largely consistent with those observed for irritable bowel syndrome. Female sex, older age, and greater disability severity were robustly associated with higher odds across all injury levels. Among regional factors, modest differences were observed by residential area, though effect sizes were small.

Physical activity showed consistent inverse associations, with all activity levels associated with lower odds of functional bowel disorder coding. Smoking and alcohol consumption were again inversely associated, but these associations were modest and should be interpreted cautiously.

Taken together, disability severity and physical activity demonstrated the most consistent and clinically interpretable associations across all injury levels.

## 4. Discussion

This nationwide study examined gastrointestinal (GI) diagnostic coding patterns among individuals with trauma-related spinal cord injury-associated healthcare encounters and identified demographic and behavioral correlates of these codes within a large administrative claims database.

Diagnostic codes for irritable bowel syndrome and other functional bowel disorders were frequently recorded, reflecting the substantial bowel symptom burden associated with neurogenic bowel dysfunction (NBD) after SCI [4,14,15]. Importantly, these codes should not be interpreted as indicating the development of new gastrointestinal diseases. Rather, within administrative healthcare data, they function as proxies for healthcare encounters related to the clinical management of bowel dysfunction in this population [4,14].

Higher odds of GI diagnostic coding among women and older adults are consistent with patterns observed in both general and SCI populations. Women may report greater symptom burden and healthcare utilization, while aging is associated with increased comorbidity, reduced mobility, and diminished bowel motility, all of which may contribute to greater symptom-driven healthcare encounters and diagnostic coding [16,17,18].

Residence in rural areas was also associated with higher odds of GI diagnostic coding. This finding may reflect disparities in healthcare access, delayed presentation, differences in service availability, or regional variation in coding practices rather than differences in underlying disease severity [19,20,21].

Disability severity demonstrated one of the most consistent associations across all GI diagnostic categories. This finding aligns with established NBD pathophysiology, whereby greater neurological impairment is associated with more severe bowel dysfunction and increased need for clinical management [4,18,22]. In addition, greater disability severity likely increases both the frequency and complexity of bowel-related healthcare encounters, thereby amplifying the likelihood of GI diagnostic code assignment within administrative data.

Inverse associations observed for smoking and alcohol consumption should be interpreted cautiously. These findings are unlikely to represent biologically protective effects and more plausibly reflect behavioral selection, reverse causation, or residual confounding. Prior studies have demonstrated that associations between smoking or alcohol consumption and health outcomes in observational data are often distorted by unmeasured confounding and misclassification, and purported protective effects of moderate alcohol consumption have been increasingly questioned [23,24,25].

Physical activity demonstrated consistent inverse associations across all models. Comparable patterns have been reported in claim-based and registry-based studies from other countries. In addition, administrative database studies from Europe and North America have shown high utilization of functional gastrointestinal diagnostic codes among individuals with spinal cord injury, reflecting symptom burden and healthcare engagement rather than incident gastrointestinal disease [26,27]. Prior studies have also reported strong associations between functional disability and bowel-related healthcare utilization, while behavioral factors such as smoking and alcohol consumption demonstrated weak or inconsistent associations after adjustment, suggesting potential confounding rather than causal effects [28,29]. Together, these findings support the interpretation that gastrointestinal diagnostic coding patterns observed in this study are not unique to the Korean healthcare system, but reflect broader challenges in capturing neurogenic bowel dysfunction within administrative data across diverse healthcare settings.

While causal inference cannot be established in this observational, claim-based study, these findings are consistent with prior literature suggesting that physical activity may support gastrointestinal motility and autonomic regulation in individuals with SCI [30,31]. Together with existing evidence, our results support continued emphasis on structured and individualized physical activity as a component of comprehensive bowel management strategies within SCI rehabilitation programs [4,5,32,33]. These findings are broadly consistent with current neurogenic bowel management guidelines, which emphasize individualized bowel programs, mobility, and regular physical activity as core components of SCI rehabilitation.

Notably, although several associations reached statistical significance, many effect sizes were modest and close to unity. This underscores the importance of distinguishing statistical significance from clinical relevance in large administrative datasets, where very small effects may achieve statistical significance due to sample size rather than reflecting meaningful clinical impact.

Several limitations should be considered. First, SCI was identified using an operational, claim-based definition, and ICD-10 codes capture healthcare utilization rather than clinically adjudicated diagnoses. Detailed neurological information, including injury level and completeness, was unavailable in the NHIS database, despite their central relevance to NBD severity. In addition, health behaviors were self-reported at a single time point, and residual confounding is likely. Administrative claims data are also subject to potential misclassification, including provider-dependent variation in coding practices, use of diagnostic codes for rule-out purposes, and upcoding or downcoding related to documentation or reimbursement practices. Such misclassification may have attenuated or inflated observed associations and should be considered when interpreting these findings. Nonetheless, the large sample size and standardized national dataset provide a robust framework for identifying population-level patterns in GI diagnostic coding after SCI.

In conclusion, GI diagnostic codes recorded after SCI should be interpreted within the context of neurogenic bowel dysfunction rather than as independent gastrointestinal diseases. These findings highlight the importance of individualized bowel management strategies and equitable access to rehabilitation services, particularly for individuals with greater disability or those residing in rural areas, to reduce symptom burden and improve long-term quality of life.

## 5. Conclusions

Gastrointestinal diagnostic codes are frequently recorded after spinal cord injury and, within administrative healthcare data, appear to reflect the clinical burden and management of neurogenic bowel dysfunction rather than the development of new gastrointestinal diseases. Recognition of these coding patterns may inform more tailored and individualized bowel management strategies, underscore the importance of structured rehabilitation care, and highlight the need for equitable access to rehabilitation services.

## Figures and Tables

**Table 1:** Baseline Characteristics of Participants at the Index Date, According to Injury Level.

Variables	Spinal Cord Injury								χ2(p)
	Cervical (S10–S19)		Thoracic (S20–S29)		Lumbar (S30–S39)		Total		
	N	(%)	N	(%)	N	(%)	N	(%)	
**Gender**									
Male	79,182	53.0%	88,421	63.1%	165,447	56.2%	333,050	57.0%	<0.001
Female	70,290	47.0%	51,814	36.9%	129,112	43.8%	251,216	43.0%	
**Age**									
<20 years	181	0.1%	76	0.1%	371	0.1%	628	0.1%	<0.001
20–39 years	49,529	33.1%	36,607	26.1%	90,144	30.6%	176,280	30.2%	
40–64 years	87,955	58.8%	79,958	57.0%	168,794	57.3%	336,707	57.6%	
≥65 years	11,807	7.9%	23,594	16.8%	35,250	12.0%	70,651	12.1%	
**Residence area**									
Metropolitan area	28,638	19.2%	24,510	17.5%	50,564	17.2%	103,712	17.8%	<0.001
Metropolitan city	37,414	25.0%	35,798	25.5%	77,764	26.4%	150,976	25.8%	
Provincial area	83,420	55.8%	79,927	57.0%	166,231	56.4%	329,578	56.4%	
**Disability Severity**									
Not applicable	143,611	96.1%	132,038	94.2%	281,158	95.5%	556,807	95.3%	<0.001
Mild (grade 4–6)	4455	3.0%	6214	4.4%	10,001	3.4%	20,670	3.5%	
Severe (grade 1–3)	1406	0.9%	1983	1.4%	3400	1.2%	6789	1.2%	
**BMI**									
<18.5	5155	3.4%	4402	3.1%	8901	3.0%	18,458	3.2%	<0.001
18.5–22.9	58,429	39.1%	53,066	37.8%	107,843	36.6%	219,338	37.5%	
23.0–24.9	35,992	24.1%	35,088	25.0%	72,413	24.6%	143,493	24.6%	
≥25.0	49,896	33.4%	47,679	34.0%	105,402	35.8%	202,977	34.7%	
**Smoking**									
Non-smoking	89,089	59.6%	73,096	52.1%	170,731	58.0%	332,916	57.0%	<0.001
Past smoking	21,275	14.2%	22,006	15.7%	43,523	14.8%	86,804	14.9%	
Current smoking	39,108	26.2%	45,133	32.2%	80,305	27.3%	164,546	28.2%	
**Drinking**									
<3 times a month	72,329	48.4%	63,875	45.5%	144,761	49.1%	280,965	48.1%	<0.001
4–7 times a month	35,824	24.0%	29,708	21.2%	67,535	22.9%	133,067	22.8%	
≥8 times a month	41,319	27.6%	46,652	33.3%	82,263	27.9%	170,234	29.1%	
**High intensity physical activity**									
Inactive	87,763	58.7%	81,702	58.3%	173,533	58.9%	342,998	58.7%	<0.001
Active	61,709	41.3%	58,533	41.7%	121,026	41.1%	241,268	41.3%	
**Moderate intensity physical activity**									
Inactive	79,160	53.0%	75,437	53.8%	157,092	53.3%	311,689	53.3%	<0.001
Active	70,312	47.0%	64,798	46.2%	137,467	46.7%	272,577	46.7%	
**Walking**									
Inactive	41,332	27.7%	41,299	29.4%	83,117	28.2%	165,748	28.4%	<0.001
Active	108,140	72.3%	98,936	70.6%	211,442	71.8%	418,518	71.6%	
**Total**	149,472	100.0%	140,235	100.0%	294,559	100.0%	584,266	100.0%	

**Table 2:** Frequency of Gastrointestinal Diagnostic Coding Within Five Years According to Injury Level.

Variables	Spinal Cord Injury								χ2(p)
	Cervical (S10–S19)		Thoracic (S20–S29)		Lumbar (S30–S39)		Total		
	N	(%)	N	(%)	N	(%)	N	(%)	
**Paralytic bowel obstruction** **without hernia (K56)**									
Non-occurrence	147,673	98.8%	138,202	98.5%	290,843	98.7%	576,718	98.7%	<0.001
Occurrence	1799	1.2%	2033	1.5%	3716	1.3%	7548	1.3%	
**Irritable Bowel Syndrome (K58)**									
Non-occurrence	103,369	69.2%	98,618	70.3%	206,466	70.1%	408,453	69.9%	<0.001
Occurrence	46,103	30.8%	41,617	29.7%	88,093	29.9%	175,813	30.1%	
**Other functional bowel** **disorders (K59)**									
Non-occurrence	93,410	62.5%	87,971	62.7%	185,574	63.0%	366,955	62.8%	0.003
Occurrence	56,062	37.5%	52,264	37.3%	108,985	37.0%	217,311	37.2%	
**Total**	149,472	100.0	140,235	100.0	294,559	100.0	584,266	100.0	

**Table 3:** Factors Associated with Paralytic Ileus According to Injury Level.

Variables	Paralytic Bowel Obstruction Without Hernia (K56)							
	Cervical (S10–S19)		Thoracic (S20–S29)		Lumbar (S30–S39)		Total	
	Adjusted OR (95% CI)	*p*	Adjusted OR (95% CI)	*p*	Adjusted OR (95% CI)	*p*	Adjusted OR (95% CI)	*p*
**Gender**								
Male	ref		ref		ref		ref	
Female	1.085 (0.988–1.19)	0.087	1.202 (1.1–1.314)	<0.001	1.078 (1.01–1.15)	0.023	1.099 (1.05–1.15)	<0.001
**Age**								
<20 years	ref		ref		ref		ref	
20–39 years	1.647 (0.23–11.78)	0.619	0.634 (0.088–4.577)	0.652	1.668 (0.415–6.702)	0.471	1.396 (0.522–3.737)	0.506
40–64 years	2.192 (0.307–15.657)	0.434	1.027 (0.143–7.392)	0.979	2.275 (0.567–9.132)	0.247	1.968 (0.736–5.263)	0.177
≥65 years	4.548 (0.635–32.578)	0.132	2.109 (0.293–15.189)	0.459	4.553 (1.133–18.295)	0.033	4.044 (1.511–10.82)	0.005
**Residence area**								
Metropolitan area	ref		ref		ref		ref	
Metropolitan city	0.975 (0.832–1.143)	0.754	1.095 (0.939–1.278)	0.2481	0.996 (0.892–1.113)	0.950	1.018 (0.942–1.101)	0.651
Provincial area	1.48 (1.297–1.689)	<0.001	1.556 (1.364–1.777)	<0.001	1.401 (1.273–1.541)	<0.001	1.462 (1.368–1.563)	<0.001
**Disability Severity**								
Not applicable	ref		ref		ref		ref	
Mild (grade 4–6)	1.833 (1.486–2.262)	<0.001	1.619 (1.359–1.928)	<0.001	1.76 (1.53–2.025)	<0.001	1.746 (1.585–1.924)	<0.001
Severe (grade 1–3)	2.238 (1.602–3.126)	<0.001	2.531 (1.978–3.237)	<0.001	2.11 (1.699–2.621)	<0.001	2.29 (1.979–2.65)	<0.001
**BMI**								
<18.5	ref		ref		ref		ref	
18.5–22.9	0.834 (0.657–1.058)	0.134	0.780 (0.621–0.98)	0.033	0.872 (0.73–1.041)	0.130	0.837 (0.742–0.945)	<0.001
23.0–24.9	0.8 (0.626–1.023)	0.076	0.753 (0.596–0.952)	0.018	0.773 (0.644–0.927)	0.006	0.775 (0.685–0.878)	<0.001
≥25.0	0.782 (0.614–0.995)	0.045	0.736 (0.584–0.926)	0.006	0.793 (0.664–0.948)	0.011	0.775 (0.686–0.875)	<0.001
**Smoking**								
Non-smoking	ref		ref		ref		ref	
Past smoking	0.948 (0.824–1.092)	0.462	0.889 (0.784–1.009)	0.068	0.948 (0.862–1.042)	0.264	0.939 (0.878–1.004)	0.064
Current smoking	1.077 (0.967–1.198)	0.177	0.792 (0.716–0.876)	<0.001	0.895 (0.829–0.967)	0.005	0.913 (0.865–0.962)	<0.001
**Drinking**								
<3 times a month	ref		ref		ref		ref	
4–7 times a month	0.808 (0.717–0.911)	0.001	0.628 (0.555–0.711)	<0.001	0.769 (0.706–0.837)	<0.001	0.739 (0.696–0.785)	<0.001
≥8 times a month	0.871 (0.779–0.973)	0.016	0.719 (0.65–0.795)	<0.001	0.836 (0.774–0.903)	<0.001	0.816 (0.773–0.86)	<0.001
**High intensity** **physical activity**								
Inactive	ref		ref		ref		ref	
Active	0.836 (0.76–0.921)	<0.001	0.717 (0.654–0.786)	<0.001	0.809 (0.757–0.866)	<0.001	0.79 (0.754–0.829)	<0.001
**Moderate intensity** **physical activity**								
Inactive	ref		ref		ref		ref	
Active	0.779 (0.709–0.856)	<0.001	0.751 (0.686–0.821)	<0.001	0.797 (0.746–0.851)	<0.001	0.779 (0.744–0.816)	<0.001
**Walking**								
Inactive	ref		ref		ref		ref	
Active	0.775 (0.702–0.855)	<0.001	0.722 (0.66–0.791)	<0.001	0.785 (0.733–0.841)	<0.001	0.763 (0.728–0.801)	<0.001

**Table 4:** Factors Associated with Irritable Bowel Syndrome According to Injury Level.

Variables	Irritable Bowel Syndrome (K58)							
	Cervical (S10–S19)		Thoracic (S20–S29)		Lumbar (S30–S39)		Total	
	Adjusted OR (95% CI)	*p*	Adjusted OR (95% CI)	*p*	Adjusted OR (95% CI)	*p*	Adjusted OR (95% CI)	*p*
**Gender**								
Male	ref		ref		ref		ref	
Female	1.265 (1.237–1.293)	<0.001	1.334 (1.303–1.365)	<0.001	1.293 (1.273–1.314)	<0.001	1.296 (1.282–1.311)	<0.001
**Age**								
<20 years	ref		ref		ref		ref	
20–39 years	0.928 (0.671–1.284)	0.652	1.014 (0.597–1.721)	0.959	0.802 (0.641–1.004)	0.055	0.856 (0.719–1.019)	0.080
40–64 years	1.221 (0.883–1.688)	0.228	1.438 (0.847–2.441)	0.178	1.123 (0.898–1.405)	0.310	1.178 (0.99–1.402)	0.065
≥65 years	1.489 (1.074–2.062)	0.017	1.703 (1.003–2.891)	0.049	1.316 (1.051–1.648)	0.017	1.385 (1.163–1.649)	<0.001
**Residence area**								
Metropolitan area	ref		ref		ref		ref	
Metropolitan city	1.095 (1.06–1.132)	<0.001	1.071 (1.034–1.11)	<0.001	1.075 (1.05–1.102)	<0.001	1.078 (1.06–1.097)	<0.001
Provincial area	0.978 (0.95–1.007)	0.136	0.993 (0.962–1.025)	0.656	0.957 (0.936–0.978)	<0.001	0.97 (0.955–0.985)	<0.001
**Disability Severity**								
Not applicable	ref		ref		ref		ref	
Mild (grade 4–6)	1.227 (1.153–1.307)	<0.001	1.277 (1.211–1.348)	<0.001	1.338 (1.284–1.395)	<0.001	1.292 (1.255–1.33)	<0.001
Severe (grade 1–3)	0.964 (0.86–1.081)	0.532	0.976 (0.885–1.076)	0.627	1.084 (1.008–1.166)	0.030	1.023 (0.971–1.078)	0.385
**BMI**								
<18.5	ref		ref		ref		ref	
18.5–22.9	0.989 (0.93–1.052)	0.732	1.043 (0.974–1.116)	0.229	1.007 (0.96–1.056)	0.7813	1.01 (0.977–1.043)	0.569
23.0–24.9	1.036 (0.973–1.104)	0.268	1.066 (0.995–1.143)	0.069	1.052 (1.002–1.104)	0.041	1.049 (1.015–1.085)	0.005
≥25.0	1.023 (0.961–1.088)	0.483	1.06 (0.99–1.134)	0.096	1.011 (0.964–1.06)	0.649	1.023 (0.99–1.058)	0.168
**Smoking**								
Non-smoking	ref		ref		ref		ref	
Past smoking	1 (0.969–1.033)	0.999	0.968 (0.937–1.000)	0.053	0.962 (0.941–0.985)	0.001	0.973 (0.957–0.989)	0.001
Current smoking	0.77 (0.75–0.791)	<0.001	0.737 (0.718–0.757)	<0.001	0.746 (0.732–0.76)	<0.001	0.749 (0.739–0.759)	<0.001
**Drinking**								
<3 times a month	ref		ref		ref		ref	
4–7 times a month	0.832 (0.809–0.855)	<0.001	0.821 (0.796–0.846)	<0.001	0.833 (0.816–0.85)	<0.001	0.83 (0.818–0.842)	<0.001
≥8 times a month	0.828 (0.806–0.85)	<0.001	0.81 (0.789–0.831)	<0.001	0.841 (0.825–0.857)	<0.001	0.829 (0.818–0.84)	<0.001
**High intensity** **physical activity**								
Inactive	ref		ref		ref		ref	
Active	0.932 (0.911–0.953)	<0.001	0.936 (0.914–0.958)	<0.001	0.937 (0.922–0.952)	<0.001	0.935 (0.925–0.946)	<0.001
**Moderate intensity** **physical activity**								
Inactive	ref		ref		ref		ref	
Active	0.924 (0.904–0.944)	<0.001	0.931 (0.91–0.953)	<0.001	0.935 (0.921–0.95)	<0.001	0.932 (0.921–0.942)	<0.001
**Walking**								
Inactive	ref		ref		ref		ref	
Active	0.922 (0.9–0.945)	<0.001	0.921 (0.898–0.944)	<0.001	0.939 (0.923–0.956)	<0.001	0.931 (0.919–0.942)	<0.001

**Table 5:** Factors Associated with Other Functional Bowel Disorders According to Injury Level.

Variables	Other Functional Bowel Disorders (K59)							
	Cervical (S10–S19)		Thoracic (S20–S29)		Lumbar (S30–S39)		Total	
	Adjusted OR (95% CI)	*p*	Adjusted OR (95% CI)	*p*	Adjusted OR (95% CI)	*p*	Adjusted OR (95% CI)	*p*
**Gender**								
Male	ref		ref		ref		ref	
Female	1.461 (1.43–1.492)	<0.001	1.547 (1.513–1.582)	<0.001	1.502 (1.479–1.524)	<0.001	1.498 (1.482–1.514)	<0.001
**Age**								
<20 years	ref		ref		ref		ref	
20–39 years	0.967 (0.708–1.32)	0.832	1.041 (0.629–1.722)	0.875	0.962 (0.771–1.2)	0.731	0.966 (0.815–1.144)	0.687
40–64 years	1.31 (0.96–1.788)	0.089	1.526 (0.923–2.524)	0.099	1.365 (1.094–1.702)	0.006	1.358 (1.147–1.609)	<0.001
≥65 years	2.223 (1.626–3.04)	<0.001	2.894 (1.749–4.787)	<0.001	2.423 (1.942–3.024)	<0.001	2.427 (2.048–2.877)	<0.001
**Residence area**								
Metropolitan area	ref		ref		ref		ref	
Metropolitan city	0.974 (0.944–1.005)	0.104	0.967 (0.935–1)	0.051	0.954 (0.932–0.977)	<0.001	0.962 (0.946–0.978)	<0.001
Provincial area	0.983 (0.956–1.011)	0.222	1.023 (0.993–1.053)	0.140	0.967 (0.947–0.987)	0.001	0.984 (0.97–0.998)	0.027
**Disability Severity**								
Not applicable	ref		ref		ref		ref	
Mild (grade 4–6)	1.468 (1.383–1.559)	<0.001	1.586 (1.507–1.669)	<0.001	1.567 (1.505–1.630)	<0.001	1.549 (1.507–1.593)	<0.001
Severe (grade 1–3)	1.524 (1.372–1.693)	<0.001	1.522 (1.392–1.663)	<0.001	1.423 (1.33–1.523)	<0.001	1.47 (1.401–1.543)	<0.001
**BMI**								
<18.5	ref		ref		ref		ref	
18.5–22.9	0.981 (0.925–1.04)	0.518	0.97 (0.91–1.033)	0.341	0.961 (0.919–1.004)	0.078	0.968 (0.938–0.998)	0.039
23.0–24.9	0.993 (0.935–1.055)	0.820	0.997 (0.935–1.064)	0.938	0.996 (0.951–1.042)	0.850	0.995 (0.964–1.027)	0.748
≥25.0	0.984 (0.928–1.044)	0.602	1.027 (0.963–1.095)	0.416	1.001 (0.958–1.047)	0.948	1.002 (0.972–1.034)	0.879
**Smoking**								
Non-smoking	ref		ref		ref		ref	
Past smoking	0.877 (0.85–0.905)	<0.001	0.873 (0.847–0.901)	<0.001	0.872 (0.853–0.891)	<0.001	0.875 (0.861–0.888)	<0.001
Current smoking	0.726 (0.708–0.744)	<0.001	0.676 (0.659–0.693)	<0.001	0.696 (0.684–0.709)	<0.001	0.699 (0.69–0.708)	<0.001
**Drinking**								
<3 times a month	ref		ref		ref		ref	
4–7 times a month	0.777 (0.757–0.798)	<0.001	0.705 (0.685–0.725)	<0.001	0.746 (0.732–0.761)	<0.001	0.745 (0.735–0.755)	<0.001
≥8 times a month	0.786 (0.766–0.806)	<0.001	0.713 (0.695–0.731)	<0.001	0.752 (0.739–0.765)	<0.001	0.751 (0.741–0.76)	<0.001
**High intensity** **physical activity**								
Inactive	ref		ref		ref		ref	
Active	0.862 (0.844–0.881)	<0.001	0.825 (0.807–0.844)	<0.001	0.857 (0.844–0.87)	<0.001	0.851 (0.841–0.86)	<0.001
**Moderate intensity** **physical activity**								
Inactive	ref		ref		ref		ref	
Active	0.874 (0.856–0.893)	<0.001	0.837 (0.819–0.856)	<0.001	0.869 (0.856–0.883)	<0.001	0.863 (0.854–0.872)	<0.001
**Walking**								
Inactive	ref		ref		ref		ref	
Active	0.899 (0.879–0.921)	<0.001	0.849 (0.829–0.87)	<0.001	0.885 (0.871–0.9)	<0.001	0.88 (0.87–0.89)	<0.001

## Data Availability

The data used in this retrospective study were accessed for research purposes on 14 March 2024 from the National Health Insurance Service (NHIS) database. All data were fully anonymized before being provided to the investigators. The authors did not have access to any information that could identify individual participants during or after data collection.

## Abbreviations

The following abbreviations are used in this manuscript:

NBD	Neurogenic bowel dysfunction
SCI	Spinal cord injury
GI	Gastrointestinal
IBS	Irritable bowel syndrome
NHIS	National health insurance service
BMI	Body mass index
KCD	Korean standard classification of diseases
